# Psyllium husk gel used as an alternative and more sustainable scalding technology for wheat bread quality improvement and acrylamide reduction

**DOI:** 10.3389/fnut.2023.1277980

**Published:** 2023-10-27

**Authors:** Elena Bartkiene, Giedre Kungiene, Vytaute Starkute, Dovile Klupsaite, Egle Zokaityte, Darius Cernauskas, Egle Kamarauskiene, Fatih Özogul, João Miguel Rocha

**Affiliations:** ^1^Department of Food Safety and Quality, Veterinary Academy, Lithuanian University of Health Sciences, Kaunas, Lithuania; ^2^Faculty of Animal Sciences, Institute of Animal Rearing Technologies, Lithuanian University of Health Sciences, Kaunas, Lithuania; ^3^Food Institute, Kaunas University of Technology, Kaunas, Lithuania; ^4^Faculty of Natural Sciences, Vytautas Magnus University, Kaunas, Lithuania; ^5^Department of Seafood Processing Technology, Cukurova University, Adana, Türkiye; ^6^Biotechnology Research and Application Center, Cukurova University, Adana, Türkiye; ^7^Universidade Católica Portuguesa, CBQF – Centro de Biotecnologia e Química Fina – Laboratório Associado, Escola Superior de Biotecnologia, Porto, Portugal; ^8^LEPABE—Laboratory for Process Engineering, Environment, Biotechnology and Energy, Faculty of Engineering, University of Porto, Porto, Portugal; ^9^ALiCE—Associate Laboratory in Chemical Engineering, Faculty of Engineering, University of Porto, Porto, Portugal

**Keywords:** wheat bread, psyllium husk, acrylamide, safety, overall acceptability

## Abstract

This study aimed at evaluating the influence of different amounts (5, 10, 15, 20, and 25%) of psyllium husk gel (PHG) on wheat bread (WB) characteristics – chiefly, overall acceptability (OA), porosity, specific volume (*v*), mass loss after baking (ML), shape retention coefficient, crust and crumb color coordinates, bread crumb hardness during storage, saccharides content, and acrylamide (AA) concentration. PHG was prepared by mixing 100 g of psyllium husk powder with 800 mL of warm water. It was established that the amount of psyllium husk gel is a significant factor in dough redness (a*) (*p* < 0.001). A moderate positive correlation (*r*) was found between acrylamide content in wheat bread and maltose concentration in dough (*r* = 0.567). The psyllium husk gel increased the overall acceptability and specific volume of wheat bread. Wheat bread porosity showed a moderate positive correlation with mass loss after baking (*r* = 0.567) and a strong positive correlation with texture hardness (*r* = 0.664). Lower acrylamide content was obtained in wheat bread prepared with 5, 10, 15, 20, and 25% of psyllium husk gel (1.53, 2.34, 3.80, 2.69, and 3.62 times lower than the control wheat bread, respectively). Acrylamide content showed a strong positive correlation with the porosity of wheat bread (*r* = 0.672), with crust brightness (L*), and yellowness/blueness (b*) coordinates, as well as with crumb brightness, redness, and yellowness coordinates. Overall, psyllium husk gel hydrocolloids reduced acrylamide formation in wheat bread and can be recommended for the quality improvement of wheat bread.

## 1. Introduction

Bread is a staple food product in many countries and its consumption ensures enough amounts of carbohydrates for human nutrition (1). Wheat bread (WB) prepared from refined flour lacks complex carbohydrates, and its structure quickly becomes hard and not acceptable for consumers during storage, especially when gluten is weak or amylase activity in flour is high (2, 3). Scalded flour (or scald) refers to the baking dough prepared with hot water (usually 95–98°C). It is popular in Nordic European countries due to improvements in the sensory characteristics and staling process of wheat bread crumb (4). Scalding advantages encompasses the reduction of enzyme activity and non-desirable microorganisms present in flours, the increase in the concentration of extractable saccharides, and the increase of the sweet taste of bread without the addition of saccharose (5–9). This technology is used at the artisanal and industrial levels but has limited application because of the requirement of hot water and its associated economic implications (5, 10). Moreover, our previous studies showed that, on the one hand, there are many advantages of adding scalded flour to the main bread formula, however, on the other hand, it can also lead to higher acrylamide (AA) concentration in the final bread (5, 6, 9). AA is present in heat-processed foods (e.g., potatoes, rice, cereal products, fruits, vegetables, meat, fish, nuts, cocoa-based products, and coffee) at various concentrations (5, 6, 11–26). Acrylamide is a Group 2A-probable human carcinogen (27), therefore the development and application of mitigation strategies are very important, especially in popular food products such as bread (28). The Maillard reaction is primarily responsible for the generation of AA, which involves condensation of reducing sugars with free amino acid asparagine, further dehydration with the Schiff base, rearrangement to the Amadori compounds or formation of azomethine, and deamination of 3-aminopropionamide (26, 29). AA is also produced when acrylic acid, which is formed from acrolein or during Maillard reaction from aspartic acid, interacts with ammonia, produced during protein breakdown (30). It was reported that AA concentration varies in cereal-based products, and these differences are related to factors such as the content of precursors (free asparagine, glucose, fructose, and maltose), the food matrix, moisture content, and the type and duration of the thermal process (baking) (31). The strategies for reducing AA in bread were reported in various studies and mainly relate to ingredients (usage of asparaginase, polyvalent cations, antioxidants, ammonium salts, and glycine; reduction of free asparagine and reducing sugars via crop’s type, harvest, and storage conditions) and processing conditions (water activity, usage of yeast or sourdough, baking time, and temperature) (29). According to the European Food Safety Authorization (EFSA) scientific opinion, coffee and its substitutes contain the highest level of AA but the category “potato fried product” is the largest contributor to total dietary exposure to acrylamide (11). EFSA has estimated the acrylamide dietary exposure of 0.5–1.9 μg/kg b.w. per day for children, and 0.4–0.9 μg/kg b.w. per day for adults and the elderly (11), while the Joint FAO/WHO Expert Committee on Food Additives (JECFA) has reported average and high dietary acrylamide exposure of 1 and 4 μg/kg/day, respectively (29). Moreover, the FoodDrinkEurope ‘Acrylamide Toolbox’ provides the industry with scientific knowledge of AA formation and its prevention and reduction in foods (32). So far, there is no safe lowest label for acrylamide (33), although this compound concentration should be as low as reasonably achievable (ALARA) (34). The European Union Regulation Commission (EU) 2017/2158 has set the benchmark levels of 50, 100, 350, and 800 μg/kg for soft bread (wheat-based), soft bread (other than wheat), crispbread, and gingerbread, respectively (34). Therefore, taking into consideration that during flour scalding higher concentrations of fermentable sugars can be obtained and these changes can lead to an elevated Maillard reaction (5), innovative solutions should be sought and integrated, especially with regard to wheat bread manufacture – when sourdough fermentation with lactic acid bacteria (LAB) is not employed as a mean to avoid acrylamide formation in the end product.

Psyllium (*Plantago ovata*) plant grows in most parts of the world (15, 16) and its health benefits are widely recognized (35–43). The soluble and insoluble dietary fiber of psyllium husk possesses very good gel-forming and high water absorption ability capacities (44, 45). The major compounds of psyllium husk are pectin, cellulose, gum, lignin, and mucilage (46). Psyllium husk arabinoxylans consist of various monosaccharides as well as hydroxyl groups, which are responsible for water absorption and psyllium husk gel properties (36, 39, 40, 43, 45, 47–49).

Psyllium husk is a well-known ingredient for gluten-free bread production but the incorporation of this hydrocolloid changes the organoleptic and technological characteristics of bread (35, 50–53). However, studies on the application of psyllium husk in white bread are scarce (1, 54–56), and there are no data reporting the influence of psyllium husk on acrylamide formation in wheat bread. The main hypothesis of this study lies in the fact that psyllium husk gel (PHG) can be used as an alternative and more sustainable solution to change the scalded flour technology toward safer wheat bread. Therefore, this study aimed to evaluate the influence of different quantities (5, 10, 15, 20, and 25%) of psyllium husk gel on wheat bread quality (porosity, specific volume, mass loss after baking, shape retention coefficient, crust and crumb color coordinates, overall acceptability, and bread crumb hardness during storage) and safety characteristic (acrylamide content). Psyllium husk gel was obtained by mixing psyllium husk powder with 30°C water. Moreover, sugar (fructose, glucose, sucrose, and maltose) content in dough and bread was determined in order to better understand the changes in Maillard reaction precursors during the technological process and their influence on acrylamide formation in produced bread.

## 2. Materials and methods

### 2.1. Materials used for breadmaking

Wheat flour (type 550D, falling number of 350 s, 27% gluten, and 0.68% ash) obtained from Kauno Grudai Ltd. mill (Kaunas, Lithuania) was used for wheat breadmaking. The wheat bread samples were prepared without and with the addition of psyllium husk gel in amounts of 5, 10, 15, 20, and 25%. Psyllium husk (pure *Plantago ovata* in powder form; 738 kJ/176 kcal per 100 g) was obtained from JSC “*Acorus Calamus*” (Svencionys, Lithuania). Psyllium husk gel was prepared by mixing 100 g of psyllium husk powder with 800 mL of (30 ± 2°C) water.

### 2.2. Breadmaking

**Figure 1:**
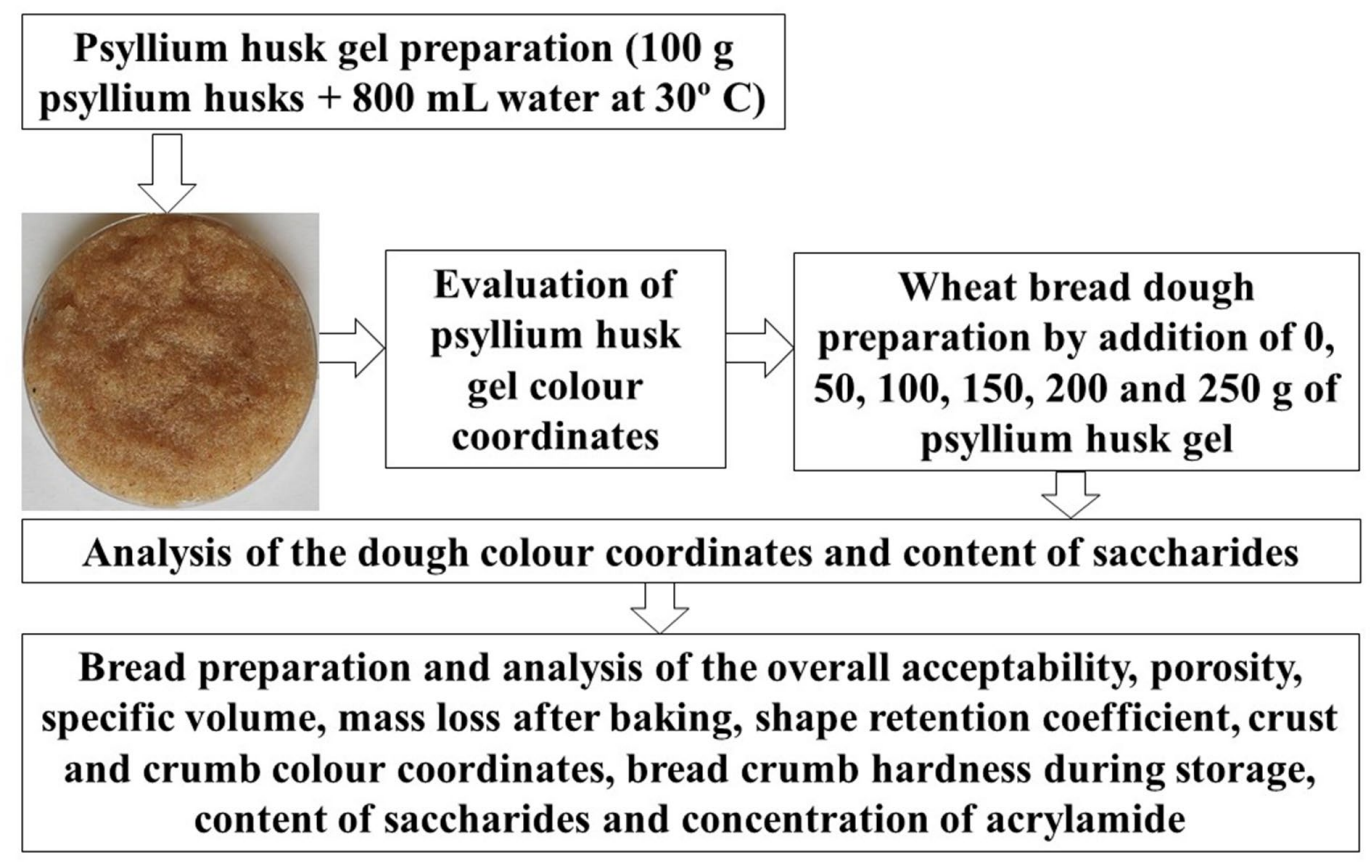
Schematic representation of the experimental design.

The bread formula consisted of 1.0 kg of wheat flour, 1.5% salt, 3% instant yeast, and 1,000 mL water (control bread). Control bread samples were prepared in the absence of psyllium husk gel. The tested dough and bread sample groups were prepared by adding 5, 10, 15, 20, and 25% (from the total flour content) of psyllium husk gel to the main recipe. Six groups of dough and bread samples were produced and examined in total. In a dough mixer (KitchenAid Artisan, Ohio, USA), the dough was blended for 2 min at a low speed, followed by 6 min at a high speed. After that, the dough was allowed to rest for 10 min at 22 ± 2°C. Following that, the dough was formed into 350 g loaves and proofed for 60 min at 30 ± 2°C and 80% relative humidity. The bread was baked for 25 min in a deck oven (EKA, Borgoricco PD, Italy) at 220°C. Figure 1 depicts a schematic illustration of the experimental design.

### 2.3. Evaluation of the psyllium husk gel and bread dough parameters

Color parameters of the psyllium husks gel and dough samples were evaluated using a CIE L*a*b* system (CromaMeter CR-400, Konica Minolta, Tokyo, Japan), where L* is brightness, a* is redness, and b* is yellowness, and, thus, −L* is darkness, −a* is greenness and-b* is blueness (57).

For sugar detection in dough, a 2 mg/mL standard solution of a sugar mixture (fructose, sucrose glucose, and maltose) was used. Analysis was performed with high-performance liquid chromatography (HPLC) and evaporative-light scattering detector (ELSD) LTII (Shimadzu Corp., Kyoto, Japan). The analysis was carried out in accordance with Klupsaite et al. (5). Supplementary File S1 has a full description of the sample preparation and procedure.

### 2.4. Evaluation of the bread quality characteristics

Bread quality characteristics were evaluated after 12 h of cooling at (22 ± 2°C). Overall acceptability of bread was carried out by 10 trained judges according to ISO 8586:2012 method (58) using a 10 score Likert scale ranging from 10 (extremely like) to 0 (extremely dislike). Additional information is given in Supplementary File S1.

Bread crumb porosity was evaluated by the Zuravliov method (LST 1442:1996). To achieve an average porosity, bread was cross-sectioned, removing the crust and crumb to form three cylinders from three distinct locations, which were then measured using the Lithuanian standard procedure (LST 1442:1996) (59). Bread volume was determined by the AACC (2003) method (60), and the specific volume was calculated as the ratio of volume to weight.

Mass loss after baking was calculated as a percentage by measuring loaf dough mass before baking and after baking. The bread shape retention coefficient was calculated as the ratio of bread slice width to height (in mm). Crust and crumb color parameters were evaluated as described in Section 2.3.

Bread crumb hardness was determined as the energy required for sample deformation (CT3 Texture Analyzer, Brookfield, USA): bread slices of 2 cm thickness were compressed to 10% of their original height at a crosshead speed of 0.5 mm/s; the resulting peak energy of compression was reported as crumb hardness. Three replicates from two different sets of baking were analyzed and averaged.

The saccharide content in bread was determined according to the method described in Section 2.3.

Acrylamide content was analyzed according to the method of Zhang et al. (61) with some modifications as described in Supplementary File S1. The compound was determined on the basis of derivatization of the target analyte with bromination. Analysis was performed with a gas chromatograph–electron capture detector (GC–ECD), using the acrylamide analytical standard.

### 2.5. Statistical analysis

The results were expressed as the mean values (for baking dough and bread samples *n* = 6, and for bread sensory characteristics and overall acceptability *n* = 10 trained panelists) ± standard error (SE). In order to evaluate the effects of different amounts of psyllium husk gel on bread quality parameters, data were analyzed using a one-way analysis of variance (ANOVA) and Tukey’s-honest significant difference (Tukey-HSD) as *post hoc* tests (statistical program R 3.2.1) (62). In addition, Pearson correlations were calculated between various parameters (63). The results were recognized as statistically significant at a *p* level equal to or below 0.05 (*p* ≤ 0.05).

## 3. Results and discussion

### 3.1. Influence of psyllium husk gel on baking dough color coordinates and concentration of saccharides

The chromaticity parameters of the psyllium husk gel and dough samples are tabulated in Table 1. Regarding the L* coordinate [brightness or (−L*) darkness], the lowest L* value was obtained in psyllium husk gel samples, and comparing among dough sample groups, different trends were found: the lowest L* value (84.4 NBS) was found in baking dough prepared with 15% of psyllium husk gel. Control baking doughs and dough groups prepared with 10 and 25% of psyllium husk gel showed similar L* values (on average, 85.6 NBS), whereas the highest L* values (on average, 86.5 NBS) were reached in baking dough groups prepared with 5 and 20% of psyllium husk gel.

**Table 1:** Chromaticity parameters of psyllium husk gel and baking dough samples.

Samples	Color coordinates		
	L*	a*	b*
	NBS		
Psyllium husk gel	52.5 ± 0.38^a^	6.81 ± 0.29^g^	17.5 ± 0.12^b^
D-C	85.6 ± 0.41^c^	−0.363 ± 0.022^a^	19.3 ± 0.14^e^
D-50	86.5 ± 0.43^d^	−0.037 ± 0.002^e^	18.3 ± 0.13^c^
D-100	85.2 ± 0.36^c^	−0.047 ± 0.003^d^	19.0 ± 0.10^d^
D-150	84.4 ± 0.28^b^	0.197 ± 0.010^b^	17.5 ± 0.12^b^
D-200	86.5 ± 0.31^d^	0.103 ± 0.009^c^	17.5 ± 0.14^b^
D-250	86.1 ± 0.45^c,d^	0.353 ± 0.021^f^	16.5 ± 0.15^a^

D – baking dough; C – control baking dough prepared without psyllium husk gel; 50, 100, 150, 200, and 250 – baking doughs prepared with 5, 10, 15, 20, and 25% psyllium husk gel, respectively; L* – brightness or (−) darkness; a* – redness or (−) greenness; b* – yellowness or (−) blueness; NBS, National Bureau of Standards units. Data are represented as means (*n* = 6) ± standard errors (SE). ^a–g^Mean values denoted with different letters indicate significantly different values between the lines (*p* ≤ 0.05).

In comparison, a* coordinate [redness or (−a*) greenness] of baking dough samples and despite the highest redness value (6.81 NBS) found in the psyllium husk gel, three out of six dough sample groups showed higher expression of greenness and then redness (control doughs and sample groups prepared with 5 and 10% of psyllium husk gel). Samples prepared with 25% of psyllium husk gel showed the highest redness coordinates (0.353 NBS), and samples prepared with 15 and 20% of psyllium husk gel presented the lowest redness coordinates (on average, 1.79 and 3.43 times lower, respectively).

Concerning the b* coordinate [yellowness or (−b*) blueness], the highest values (19.3 NBS) were observed in the control samples, whereas the other samples showed values lower than 4.5 and 1.55% (samples prepared with 25 and 10% of psyllium husk gel, respectively). Finally, tests of between-subject effects showed that the amount of psyllium husk gel is a statistically significant factor on dough a* coordinate values (*p* < 0.001).

Information about the color coordinates of psyllium husk gel or its effect on dough chromaticity properties is scarce. However, it was reported that psyllium husk powder L*, a*, and b* coordinate values were 66.64, 4.01, and 12.36 NBS, respectively (64). In addition, the data about the influence of psyllium husk on noodle dough stated that L* and b* values decrease while a* value increases by increasing its content in the dough (40). Another study revealed that psyllium husk added to sponge cake led to an increase in the dough L* and a* values and a decrease in the b* value (65). Gómez et al. testified that fiber concentration is responsible for the change in a* and b* parameters in baking doughs (66). Differences in color coordinates of tested doughs likely occurred due to the natural pigments present in psyllium husk gel and different mechanisms of its action in the baking dough, including its influence on proteins and enzyme activities. However, further, deeper research is required to evaluate the exact influence of psyllium husk gel on the chromaticity parameters of baking dough.

**Figure 2:**
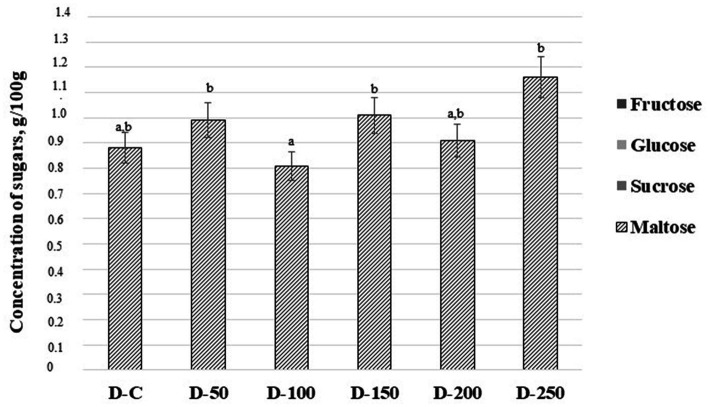
Concentration of sugars (g/100 g) in baking dough samples [D – baking dough; C – control baking dough prepared without psyllium husk gel; 50, 100, 150, 200, and 250 – baking doughs prepared with 5, 10, 15, 20, and 25% psyllium husk gel, respectively; Data are represented as means (*n* = 6) ± standard errors (SE); ^a–b^Mean values denoted with different letters indicate significantly different values between the columns (*p* ≤ 0.05)].

The concentration of sugars (g/100 g) in baking dough samples is shown in Figure 2. Among all analyzed sugars (fructose, glucose, sucrose, and maltose) only maltose could be detected in the baking dough samples. The lowest content of maltose was found in samples prepared with 10% of psyllium husk gel (0.810 g/100 g). Control samples and samples prepared with 20% of psyllium husk gel showed a slightly higher content of maltose but it was not statistically significant (on average, 0.895 g/100 g). In samples prepared with 5, 15, and 25% of psyllium husk gel, maltose content was, on average, 22.9% higher than in samples prepared with 10% of psyllium husk gel (the latter had the lowest maltose content).

The predominant saccharides found in refined wheat flour are sucrose (2.16 ± 0.26 mg/g of flour), maltose (0.53 ± 0.09 mg/g of flour), fructose (0.91 ± 0.13 mg/g of flour), and glucose (0.54 ± 0.09 mg/g of flour) (67). Sucrose is rapidly hydrolyzed by yeast into glucose and fructose (68). In addition to glucose and fructose, maltose and raffinose can be found in flour, and polyols can also be synthesized by microorganisms present in doughs (69). During breadmaking, when yeasts are used for fermentation, the fermentable saccharides are converted into carbon dioxide and ethanol (70), with a preference for glucose consumption over fructose and maltose (69). This partly explains the low profile of detected sugars in tested doughs. Moreover, the fact that arabinoxylans in psyllium husk consist mostly of arabinose and xylose, as well as minor levels of rhamnose, galactose, and glucose, also contributes to the obtained results (37). Changes in maltose content between tested doughs could be explained by the fact that psyllium husk gel as dietary fiber/hydrocolloid could interact with dough constituents and entrap them or disrupt the internal relations (71). For example, the entrapment of starch could limit its availability for enzymes and sugar release. Nevertheless, there is currently no universal agreement on the mechanism of action of such hydrocolloids (72).

### 3.2. Influence of psyllium husk gel on the main parameters of bread

**Figure 3:**
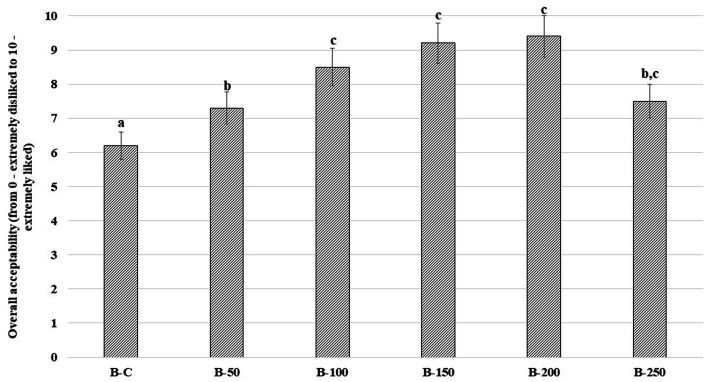
Overall acceptability (OA) of wheat bread (WB) [B – bread; C – control bread prepared without psyllium husk gel; 50, 100, 150, 200, and 250 – bread samples prepared with 5, 10, 15, 20, and 25% psyllium husk gel, respectively; Data are represented as means (*n* = 10) ± standard errors (SE); ^a–c^Mean values denoted with different letters indicate significantly different values between the columns (*p* ≤ 0.05)].

**Figure 4:**
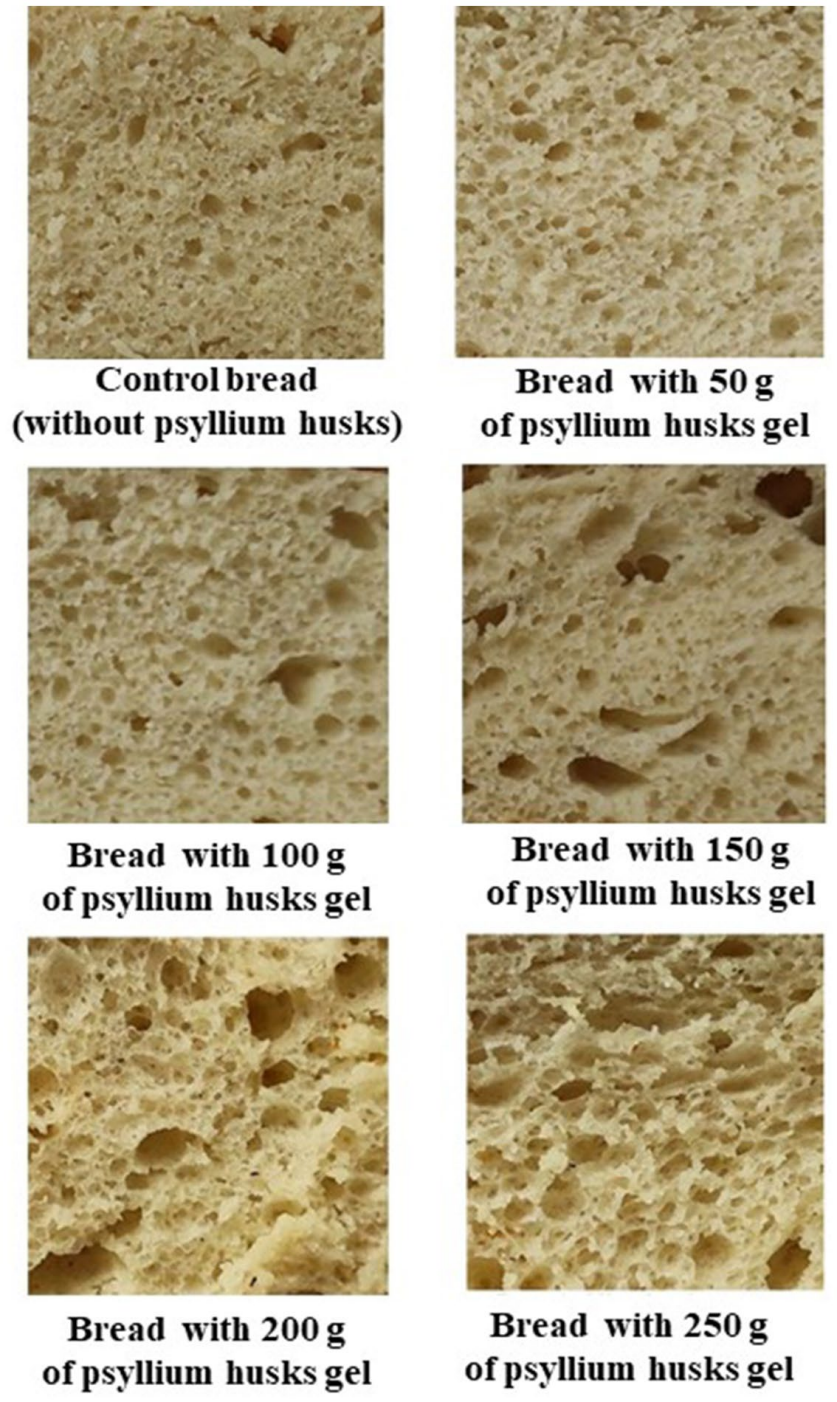
Images of wheat bread crumb samples.

Scores of the bread’s overall acceptability and bread crumb images are presented in Figures 3, 4, respectively. In comparison with control bread, sample groups prepared with psyllium husk gel showed higher overall acceptability (Figure 3), chiefly, the overall acceptability of samples with 5% of psyllium husk gel was, on average, 17.7% higher, the overall acceptability of samples with 10, 15, and 20% of psyllium husk gel was, on average, 45.6% higher, and the overall acceptability of samples with 25% of psyllium husk gel was, on average, 21.0% higher than the control.

The higher acceptability of tested bread with psyllium husk gel could be related to the softer texture and increased porosity of bread crumbs (73). Similarly to our results, it was conveyed that the incorporation of psyllium (at 2 or 5%) improved the taste of whole meal breads by increasing their overall acceptability (1). Furthermore, bread with a greater level of moisture has more juiciness, which consumers prefer, and that could also be the reason for the higher acceptability scores of tested breads in our study (57). Moriarty et al. suggested that highlighting the advantages of psyllium husk incorporation in the main bread recipes (e.g., fewer calories, higher amount of fiber, etc.), could be a lever to increase the acceptability of bread with psyllium (74). Lastly, psyllium husk gel, as an ingredient for refined wheat flour bread preparation, can increase the functional value and overall acceptability of such types of bread.

Observing the bread crumb images (Figure 4), a clear visual trend was perceived. In particular, by increasing psyllium husk gel amounts to the main bread recipe, the number of large pores in bread crumb increased. In relation to the retention coefficient of bread, samples prepared with 15 and 25% of psyllium husk gel showed lower shape retention coefficient values than the control bread and bread samples prepared with 5, 10, and 20% of psyllium husk gel (on average, 18.9% lower) (Table 2). Taking into consideration that the bread shape retention coefficient is calculated as the ratio between bread slice width and height, this means that the lower the shape retention coefficient values, the higher the height of the bread loaf. In addition, by increasing psyllium husk gel amounts to the main bread formula, the specific volume of bread increased. In comparison with control samples, bread prepared with 5 and 10% of psyllium husk gel showed, on average, 5.65% higher specific volume, bread with 15 and 20% of psyllium husk gel showed, on average, 12.3% higher specific volume, and bread with 25% of psyllium husk gel showed, on average, 16.6% higher specific volume. Similar tendencies were established for the porosity of bread samples, i.e., by increasing psyllium husk gel amounts in the main bread formula, the porosity of bread samples increased. Nevertheless, the correlation (*r*) between bread-specific volume and porosity was not statistically significant. However, bread porosity showed a moderate positive correlation with bread mass loss after baking (*r* = 0.567, *p* = 0.14).

**Table 2:** Bread quality parameters: shape retention coefficient, mass loss (ML) after baking, specific volume (*v*), and porosity.

Bread samples	Shape retention coefficient	Mass loss after baking, %	Specific volume, cm^3^/g	Porosity, %
B-C	2.15 ± 0.14^b^	6.41 ± 0.22^a^	3.01 ± 0.05^a^	70.5 ± 0.39^a^
B-50	2.23 ± 0.12^b^	7.67 ± 0.19^b^	3.14 ± 0.04^b^	72.9 ± 0.41^b^
B-100	2.33 ± 0.10^b^	8.81 ± 0.21^c^	3.22 ± 0.05^b^	74.3 ± 0.52^c^
B-150	1.87 ± 0.11^a^	9.60 ± 0.32^d^	3.34 ± 0.04^c^	75.7 ± 0.39^d^
B-200	2.07 ± 0.06^b^	10.6 ± 0.28^e^	3.41 ± 0.03^c^	77.5 ± 0.47^e^
B-250	1.69 ± 0.07^a^	11.8 ± 0.26^f^	3.51 ± 0.04^d^	78.6 ± 0.59^f^

B – bread; C – control bread prepared without psyllium husk gel; 50, 100, 150, 200, and 250 – bread samples prepared with 5, 10, 15, 20, and 25% psyllium husk gel, respectively. Data are represented as means (*n* = 6) ± standard errors (SE). ^a–f^Mean values denoted with different letters indicate significantly different values between the different bread samples.

Reported data on the effect of psyllium husk on bread quality features are inconsistent. Our results are in agreement with Yassin et al., who observed that the addition of psyllium husk significantly increased bread loaf volume in comparison with control samples (75). Conversely, the results from Man et al. showed opposite tendencies, i.e., by increasing psyllium husk amounts, loaf-specific volume decreased, with these changes being explained by the dilution of the gluten network caused by psyllium fiber (76). The research from Mironeasa and Codina also showed that the specific volume of bread decreased as the concentration of psyllium increased (55). It should be mentioned that the difference in the reported data could be due to multiple factors, e.g., different processing steps, different ingredient characteristics, etc. In our study, the higher specific volume and porosity after baking bread with psyllium husk gel could be explained by the presence of water-soluble dietary fibers, which contribute to an additional polymeric network formation within the dough and, thus, led to a stronger and more extensible viscoelastic structure that improved the gas retention capacity of dough and increased loaf specific volume (75). Moreover, a greater water binding capacity in bread dough can reinforce the gluten network, possibly by giving a better plasticizing impact and minimizing regions of extremely compact gluten-gluten intermolecular relations (77). In terms of crumb structure, it was reported that higher concentrations of psyllium husk contribute to the lessened uniformity of the crumb structures with a tendency toward larger air cells (75), and these changes in crumb structures could contribute to the different mechanical and sensory characteristics of bread (78).

**Figure 5:**
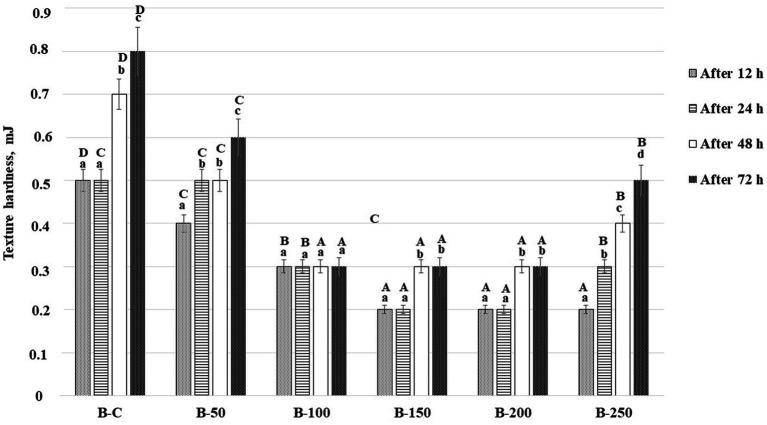
Changes in bread texture hardness (mJ) during storage [B – bread; C – control bread prepared without psyllium husk gel; 50, 100, 150, 200, and 250 – bread samples prepared with 5, 10, 15, 20, and 25% psyllium husk gel, respectively; Data are represented as means (*n* = 6) ± standard errors (SE); ^a–d^Mean values denoted with different letters indicate significantly different values between the same group of samples after different storage time period; ^A–D^Mean values denoted with different letters indicate significantly different values between the different bread samples after the same storage time period; (*p* ≤ 0.05)].

Changes in bread texture hardness during bread storage (12, 24, 48, and 72 h) are shown in Figure 5. Analyzing the bread texture hardness after 12 h of storage, it was unveiled that by increasing the psyllium husk gel amount to the main bread formula, the hardness was reduced. Moreover, in comparison with control samples, bread prepared with 5% of psyllium husk gel presented, on average, 20.0% lower crumb hardness, bread prepared with 10% of psyllium husk gel, showed, on average, 40.0% lower crumb hardness, and bread prepared with 15, 20, and 25% of psyllium husk gel disclosed, on average, 60.0% lower crumb hardness. After 24 h of storage, control samples showed hardness values similar to those obtained after 12 h of storage (0.500 mJ). However, bread prepared with 5 and 25% of psyllium husk gel showed increased hardness – on average, by 25 and 50%, respectively – when comparing samples with 12 and 24 h of storage.

In comparison with the hardness of the same group of samples after 12 h, it was observed that after 48 h of storage, the hardness of the control bread increased, on average, by 1.4 times, the hardness of bread with 5% of psyllium husk gel increased, on average, by 1.25 times; that of bread with 10% of psyllium husk gel, remained similar, that of bread with 15 and 20% of psyllium husk gel increased, on average, by 1.5 times, and the hardness of bread with 25% of psyllium husk gel increased, on average, by 2.0 times. It is important to underline that despite the higher changes observed in bread prepared with 15, 20, and 25% of psyllium husk, their texture hardness after 48 h of storage was lower, on average, by 57.1% (bread with 15 and 20% of psyllium husk gel) and 42.9% (bread with 25% of psyllium husk gel), in comparison with the control bread samples. After 72 h of storage, similar tendencies were seen. In all cases, the hardest structure was obtained in the control samples and the lowest hardness was found on bread prepared with 10, 15, and 20% of psyllium husk gel (on average, 0.300 mJ).

Usually, during storage, the bread hardness increases because of the starch retrogradation process. However, the staling process is slower in bread prepared with high water-binding capacity components (75). In breadmaking, psyllium husk is used as a technological improver, particularly to slow bread staling by changing and delaying the starch retrogradation process in wheat bread (50, 79). In our study, the softer bread texture during storage could be explained by PHG’s ability to retain moisture and the higher specific volume of bread (75, 80). Moreover, the anti-staling effect of PHG could be explained by its fiber’s hydrogen bonding to starch amylopectin, which inhibits the formation of crystalline structures (81). It was reported that psyllium husk improves the quality of gluten-free bread, including its quality during storage (82). Abdullah et al. found out that bread enriched with 5% of psyllium husk showed a softer texture, in comparison with controls (57). However, another study revealed that these changes are related to the bread formulation and the addition of 5% of psyllium husk to white wheat flour significantly softened the bun texture, whereas, the addition of the same quantity of psyllium husk to whole wheat flour resulted in a much harder texture of the bun (57). Yassin et al. reported that the non-starch polysaccharides lower bread hardness (75). Our results are in agreement with the abovementioned findings. Moreover, a strong positive correlation between the bread texture hardness (after 12 h of storage) and the porosity of the samples (*r* = 0.664, *p* = 0.003) was found in our study. Usually, higher porosity leads to a faster bread staling process due to faster water migration from the bread crumb. However, in this study, the obtained results showed the opposite, and it can be stated that hydrocolloids, in addition to the increased bread porosity, have a lowering effect on water migration in bread crumb and lead to a softer bread during storage.

### 3.3. Concentration of acrylamide in bread and its relation with bread crust and crumb chromaticity characteristics and content of sugars

**Figure 6:**
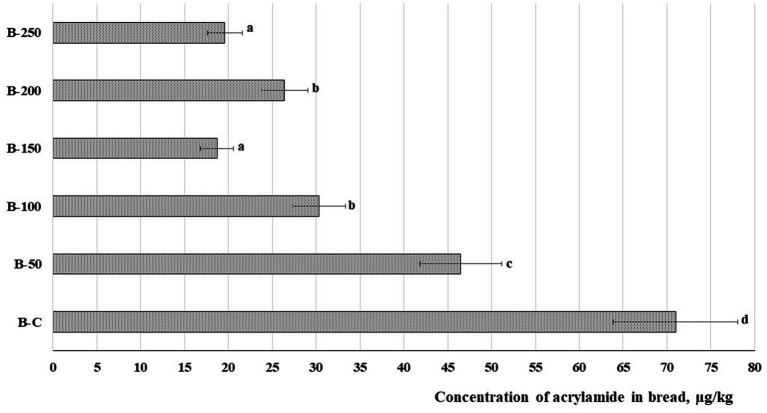
Concentration of acrylamide (μg/kg) in bread [B – bread; C – control bread prepared without psyllium husk gel; 50, 100, 150, 200, and 250 – bread samples prepared with 5, 10, 15, 20, and 25% psyllium husk gel, respectively; Data are represented as means (*n* = 6) ± standard errors (SE). ^a–d^Mean values denoted with different letters indicate significantly different values between the different bread samples (*p* ≤ 0.05)].

Acrylamide concentration in bread samples is given in Figure 6. It is apparent in all cases that a lower content of acrylamide was obtained in bread prepared with psyllium husk gel. Samples prepared with 5, 10, 15, 20, and 25% of psyllium husk showed, respectively, on average, 1.53, 2.34, 3.80, 2.69, and 3.62 times lower acrylamide concentration than the control bread. A strong positive correlation was found between acrylamide content and the porosity of the bread (*r* = 0.672, *p* = 0.002). Yet, a moderate positive correlation was established between acrylamide content in bread and maltose concentration in dough samples (*r* = 0.567, *p* = 0.014) (Table 3).

**Table 3:** Pearson correlations (*r*) between the content of acrylamide in bread and bread crumb and crust color coordinates, overall acceptability (OA), mass loss (ML) after baking, specific volume (*v*), porosity, and content of sugars in bread, and their significance (*p*).

Bread quality parameters	Acrylamide	
	*r*	*p*
L* crust	0.753**	<0.001
a* crust	0.264	0.289
b* crust	0.707**	0.001
L* crumb	0.657**	0.003
a* crumb	−0.684**	0.002
b* crumb	0.530*	0.024
Overall acceptability	−0.460	0.054
Mass loss after baking	0.194	0.441
Specific volume	−0.183	0.468
Porosity	0.672**	0.002
Maltose content in dough	0.567*	0.014
Maltose content in bread	−0.049	0.848
Fructose content in bread	0.312	0.208

L* – brightness or (−) darkness; a* – redness or (−) greenness; b* – yellowness or (−) blueness. *r*, Pearson correlation; *p*, significance. **Correlation is significant at the 0.01 level (2-tailed). *Correlation is significant at the 0.05 level (2-tailed).

The chromaticity parameters of bread crust and crumb are summarized in Table 4. Observing the L* values of bread crust, samples prepared with 15 and 25% of psyllium husk gel showed, respectively, on average, 3.50 and 12.9% lower L* coordinates than the control bread. In addition, bread prepared with 5, 10, and 20% of psyllium husk showed, respectively, on average, 20.3, 6.43, and 4.42% higher L* values than the control samples. A strong positive correlation was found between acrylamide content in samples and bread crust L* coordinate values (*r* = 0.753, *p* < 0.001) (Table 3).

**Table 4:** Chromaticity (L*, a*, and b*) parameters in bread crust and crumb.

Samples	Color coordinates		
	L*	a*	b*
B-Ccrust	54.3 ± 0.51^c^	9.98 ± 0.09^b^	22.7 ± 0.21^b^
Bcrust-50	65.3 ± 0.52^f^	6.52 ± 0.21^a^	22.4 ± 0.22^b^
Bcrust-100	57.8 ± 0.48^e^	11.8 ± 0.11^e^	28.3 ± 0.23^e^
Bcrust-150	52.4 ± 0.46^b^	10.5 ± 0.09^c^	24.1 ± 0.20^c^
Bcrust-200	56.7 ± 0.33^d^	10.8 ± 0.07^d^	24.7 ± 0.21^d^
Bcrust-250	47.3 ± 0.32^a^	21.3 ± 0.12^f^	21.3 ± 0.19^a^
B-Ccrumb	72.9 ± 0.47^a^	−0.780 ± 0.019^a^	21.3 ± 0.17^e^
Bcrumb-50	73.8 ± 0.39^b,c^	−0.630 ± 0.022^b^	21.1 ± 0.20^e^
Bcrumb-100	74.5 ± 0.37^c^	−0.451 ± 0.024^d^	19.4 ± 0.17^d^
Bcrumb-150	74.4 ± 0.32^c^	−0.551 ± 0.015^c^	18.7 ± 0.16^c^
Bcrumb-200	73.3 ± 0.40^b^	−0.472 ± 0.017^d^	18.0 ± 0.14^b^
Bcrumb-250	72.4 ± 0.39^a^	−0.090 ± 0.008^e^	17.3 ± 0.13^a^

B, bread; C, control bread prepared without psyllium husk gel; 50, 100, 150, 200, and 250 – bread samples prepared with 5, 10, 15, 20, and 25% psyllium husk gel, respectively; L* – brightness or (−) darkness; a* – redness or (−) greenness; b* – yellowness or (−) blueness; NBS – National Bureau of Standards units. Data are represented as means (*n* = 6) ± standard errors (SE). ^a–f^Mean values denoted with different letters indicate significantly different values between the different bread crust and crumb samples.

Regarding bread crust a* values, samples prepared with 5% of psyllium husk gel showed, on average, 34.7% lower a* coordinates than the control breads. However, other bread groups prepared with psyllium husk gel, presented significantly higher a* values than the control bread crust samples. The lowest crust b* coordinates (21.3 NBS) were found in bread prepared with 25% of psyllium husk gel. The b* coordinates were, on average, 22.6 NBS in the control bread and samples prepared with 5% of psyllium husk gel. Other bread crust samples (*viz.*, with 10, 15, and 20% of psyllium husk gel) showed, respectively, on average, 25.2, 6.64, and 9.29% higher b* values than the control bread and bread prepared with 5% of psyllium husk gel. A strong positive correlation was attained between acrylamide content and bread and bread crust b* values (*r* = 0.707, *p* = 0.001) (Table 3). In comparison, bread crumb color coordinates and the lowest L* values were achieved in control samples and samples prepared with 25% of psyllium husk gel (on, average, 72.7 NBS), whereas the lowest a* coordinates were found in control samples (−0.780 NBS), and the lowest b* values were established in bread prepared with 25% of psyllium husk gel (17.3 NBS). A strong positive correlation was obtained between acrylamide content in bread samples and bread crumb L* values (*r* = 0.657, *p* = 0.003), a strong negative correlation was established between acrylamide content in bread samples and bread crumb a* values (*r* = −0.684, *p* = 0.002), and a moderate positive correlation was obtained between acrylamide content in bread samples and bread crumb b* coordinates (*r* = 0.530, *p* = 0.024) (Table 3).

Surdyk et al. reported a strong correlation between acrylamide content in bread and crust color (83). Likewise, Dessev et al. indicated a strong positive correlation between total color difference and acrylamide content in bread (84).

**Figure 7:**
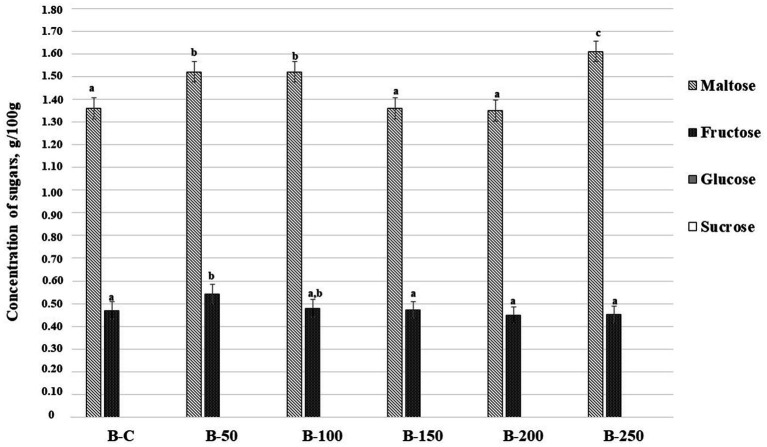
Concentration of sugars (g/100 g) in bread samples [B – bread; C – control bread prepared without psyllium husk gel; 50, 100, 150, 200, and 250 – bread samples prepared with 5, 10, 15, 20, and 25% psyllium husk gel, respectively; Data are represented as means (*n* = 6) ± standard errors; ^a–c^Mean values denoted with different letters indicate significantly different values between the different sugars (*p* ≤ 0.05)].

The concentration of sugars (g/100 g) in bread samples is depicted in Figure 7. From all analyzed sugars (fructose, glucose, sucrose, and maltose) only maltose and fructose were found in bread samples. Maltose content in bread samples varied and the highest concentration was established in bread prepared with 25% of psyllium husk gel (1.61 g/100 g). The highest concentration of fructose was quantified in bread prepared with 5% of psyllium husk gel (0.541 g/100 g). It is worth noting that fructose content was slightly lower in samples prepared with 10% of psyllium husk gel but it was not statistically different. Moreover, significant correlations between acrylamide and the content in bread samples of analyzed sugars were not established (Table 3).

The reduction or dilution of acrylamide precursors in baking dough is considered a prospective strategy to decrease acrylamide formation in the final bread (29). Maillard reaction, as a non-enzymatic browning, is involved with desirable properties in cereal-based products, chiefly color and aroma (85). During the first stages of acrylamide formation, the interaction between carbonyl and amino groups results in Schiff bases (29). The latter rearrange to Amadori or Heyns products (86). However, the Schiff bases may be decarboxylated or hydrolyzed and form azomethine ylide as well as 3-aminopropionamide. Additionally, acrylamide may be formed via deamination of 3-aminopropionamide or directly by azomethine ylide (87, 88). Constituents of the flour play major roles in acrylamide formation, and acrylamide is formed in higher concentrations in bread manufactured with high extraction rate flours (89). For this reason, improving the functional value of bread by incorporating the outer layer of cereal dietary fiber becomes very challenging and, to overcome related difficulties, new ingredients such as psyllium husks can be very promising. Additionally, to reduce acrylamide concentration in bread, technological solutions such as reducing baking temperatures and, simultaneously, prolonging the baking process may be suggested, because high temperatures and low water activities are the major factors favoring acrylamide formation (90). However, during baking, crumb and crust temperatures of bread are different; as a reference, at the end of the process, the crust, on average, reaches 200°C, whereas the crumb reaches, on average, 98°C. For this reason, the crust shows a lower water activity than the crumb and, consequently, acrylamide concentration in the crumb is much lower than in the crust (91). Moreover, baking time and temperature are related to the rate of water loss from the surface of the bread (92), as well as higher bread porosity being able to lead to more water migration. Nevertheless, the brown color and crispy texture of the crust are important characteristics in the overall acceptability of bread (90) and, for this reason, in the current study, acrylamide analysis of bread loaf was determined to better meet a strategy for bread consumption.

Despite the temperature of the crumb being much lower than in the crust, Maillard and caramelization reactions during baking are the main factors for the formation of colors in bread crumb, although variations in color may be related to different contents of distinct pigments (93). Additionally, a compacted structure of crumb leads to lower lightness with a simultaneous increase in redness and yellowness (94). Our current research showed that there is a strong positive correlation between acrylamide content in samples and the porosity of the bread (Table 3). Despite that there are many recommendations for acrylamide reduction in bread, by reducing temperature and prolonging the duration of the baking process (30, 83, 84, 95–102), it should be highlighted that these technological solutions can lead to lower acceptability of the final baking products because of the low intensity of the crust color. The latter characteristic depends strongly on the temperature: the higher the surface temperature, the higher the moisture loss of the surface layers, and the more effective the Maillard reaction, thus, resulting in the darker color of the bread crust (103). Evaporation of water is related to the dough temperature reached during baking, and when any part of the baking dough reaches a temperature significantly above the boiling point, its water content becomes so low that evaporation is restricted and the balance between heat absorption and evaporation loss is altered. After this stage, the process of crust formation starts (84). However, our study showed that psyllium husk gel increases the softness of the bread and it is involved in the reduction of the moisture migration process of bread samples with higher porosity (bread porosity showed moderate positive correlation with bread mass loss after baking). Finally, in accordance with the lower acrylamide concentration obtained in bread prepared with psyllium husk gel, it can be stated that the psyllium husk gel hydrocolloids reduce water migration from bread during baking and, at the same time, reduce acrylamide formation in the end products.

## 4. Conclusion

This research paper provides an application of different quantities of psyllium husk gel to wheat bread production and evaluates its effect on bread quality and the formation of acrylamide, which is a neurotoxic and carcinogenic compound. Results revealed that psyllium husk gel improved the overall acceptability of the wheat bread from refined flour, and by increasing the amount of psyllium husk gel in the main bread recipe, specific volume and porosity increased. Bread containing psyllium husk gel had a softer crumb in all cases. Moreover, positive correlations were identified between porosity and mass loss after baking or texture hardness. Although the higher porosity of the bread usually leads to a faster staling process due to the faster water migration from the crumb, the results of our study were the opposite. Psyllium husk gel hydrocolloids showed, in addition to an increased bread porosity, a reducing effect on water migration from crumb and a softer texture during bread storage. Acrylamide formation was significantly diminished by the addition of psyllium husk gel to wheat bread due to its ability to reduce water migration during baking. The outcome of this study opens avenues for the application of psyllium husk gel in obtaining safer and higher-quality wheat bread.

## Data availability statement

The original contributions presented in the study are included in the article/Supplementary material, further inquiries can be directed to the corresponding author.

## Author contributions

EB: Conceptualization, Project administration, Supervision, Writing – original draft, Writing – review & editing. GK: Formal analysis, Investigation, Writing – original draft. VS: Data curation, Investigation, Methodology, Validation, Writing – original draft. DK: Formal analysis, Investigation, Methodology, Writing – original draft. EZ: Formal analysis, Software, Writing – original draft. DC: Formal analysis, Writing – original draft. EK: Data curation, Investigation, Writing – original draft. FÖ: Investigation, Writing – review & editing. JR: Project administration, Writing – review & editing.

## Glossary

AA: Acrylamide
ANOVA: Analysis of variance
*r*: Correlation
CH_3_COOC_2_H_5_: Ethyl acetate
ELSD: Evaporative-light scattering detector
GC–ECD: Gas chromatograph – electron capture detector
HPLC: High-performance liquid chromatography
LAB: Lactic acid bacteria
ML: Mass loss after baking
NBS: National Bureau of Standards units
OA: Overall acceptability
KBrO_3_: Potassium bromate
KBr: Potassium bromide
PHG: Psyllium husk gel
NaCl: Sodium chloride
Na_2_SO_4_: Sodium sulfate
Na_2_S_2_O_3_x5H_2_O: Sodium thiosulfate pentahydrate
*v*: Specific volume
SE: Standard error
H_2_SO_4_: Sulfuric acid
(C_2_H_5_)3N: Triethylamine
Tukey-HSD: Tukey’s-honest significant difference
WB: Wheat bread
