# Supplementary material for: Psyllium husk gel used as an alternative and more sustainable scalding technology for wheat bread quality improvement and acrylamide reduction
Source: Front Nutr. 2023 Oct 27;10:1277980. doi: 10.3389/fnut.2023.1277980 (PMC10641513; doi:10.3389/fnut.2023.1277980)
Supplement: Supplementary file 1 [file Data_Sheet_1.PDF]

Supplementary Material

1. Evaluation of the bread overall acceptability

Bread quality characteristics were evaluated after 12 h of cooling at room temperature (22±2 °C). Overall acceptability of breads was carried out by 10 trained judges according to ISO 8586:2023 method (1) using a 10 scores Likert scale ranging from 10 (extremely like) to 0 (extremely dislike). Ten selected judges from the internal recruitment (Department of Food Safety and Quality and Institute of Animal Rearing Technologies, Lithuanian University of Health Sciences, Lithuania, Kaunas) were chosen (2,3). The previous training of the assessors was based on the descriptive analysis (4,5). Selected assessors were no smoking persons, interested in sensory analysis and motivated.

References

1. ISO 8586 (2012) Sensory Analysis — General Guidelines for the Selection, Training and Monitoring of Selected Assessors and Expert Sensory Assessors (Standard revised in 2023); Available online: https://www.iso.org/cms/render/live/en/sites/isoorg/contents/data/standard/04/53/45352.html (accessed on 21 August 2023).

2. Standardization, I.O. for ISO 5492:2008. Sens Anal; 2008.

3. Torres, F.R.; Esmerino, E.A.; Carr, B.T.; Ferrão, L.L.; Granato, D.; Pimentel, T.C.; Bolini, H.M.A.; Freitas, M.Q.; Cruz, A.G. Rapid Consumer-Based Sensory Characterization of Requeijão Cremoso, a Spreadable Processed Cheese: Performance of New Statistical Approaches to Evaluate Check-All-That-Apply Data. J Dairy Sci 2017; 100, 6100–6110, doi:10.3168/jds.2016- 12516.

4. Gaze, L.V.; Oliveira, B.R.; Ferrao, L.L.; Granato, D.; Cavalcanti, R.N.; Conte Júnior, C.A.; Cruz, A.G.; Freitas, M.Q. Preference Mapping of Dulce de Leche Commercialized in Brazilian Markets. J Dairy Sci 2015; 98, 1443–1454, doi:10.3168/jds.2014-8470.

5. Janiaski, D.R.; Pimentel, T.C.; Cruz, A.G.; Prudencio, S.H. Strawberry-Flavored Yogurts and Whey Beverages: What Is the Sensory Profile of the Ideal Product? J Dairy Sci 2016; 99, 5273– 5283, doi:10.3168/jds.2015-10097.

2. Sugars analysis

To determine the sugar concentration in dough, 1–2 g of sample was diluted in 60 mL of distilled/deionized water, heated to 60 °C in a water bath for 15 min, clarified with 2.5 mL Carrez I {85 mM $K_4[Fe(CN)_6] \times 3H_2O$} and 2.5 mL Carrez II (250 mM $ZnSO_4 \times 7H_2O$) solutions, and made up to 100 mL with distilled/deionized water. After 15 min, samples were filtered through a filter paper and a 0.22 μm-ϕ nylon syringe filter before further analysis. A 2 mg/mL standard solution of a sugar's mixture was prepared by dissolving 0.2 g of each of fructose, glucose, sucrose and maltose (Sigma-Aldrich, Hamburg, Germany) in 100 mL of distilled/deionized water. High-performance liquid chromatography (HPLC) conditions were

as follows: the eluent was a mixture of 75 parts by volume of acetonitrile and 25 parts by volume of water, the flow-rate was 1.2 mL/min, and 20 µL of the extract was injected. A YMC-Pack Polyamine II 250 × 4.6 mm-ϕ, 5 µm-ϕ particle size column (YMC Co., Ltd., Tokyo, Japan) was used. The column temperature was set at 28 °C. The detection was performed using an Evaporative-light scattering detector (ELSD) LTII (Shimadzu Corp., Kyoto, Japan.

3. Acrylamide evaluation in bread

The bread samples were homogenized in a blender (Ika A10, Germany). Two grams of sample were weighed in 50 mL centrifuge tube and diluted with 20 mL of distilled/deionized water. Sampling tube was briefly vortexed (ZX3 Advanced VELP, Italy) for 10 min. The sample tube was centrifuged (Hermle Z 306, Germany) at 4,000 rpm for 10 min. Ten millilitres of the clarified aqueous layer solution in 15 mL centrifuge tubes were clarified with 100 µl of Carrez I {85 mM $K_4[Fe(CN)_6] \times 3H_2O$} and 100 µl of Carrez II (250 mM $ZnSO_4 \times 7H_2O$) solutions. The sample tubes were, then, centrifuged at 4,000 rpm for 10 min. For the preparation of acrylamide standard solution (30.4 µg/L), 15.2 mg of acrylamide analytical standard (99.8% purity) was weighed and dissolved in a 1000 mL volumetric flask and diluted with deionized water. The obtained solution was diluted by pouring 2 mL of the obtained acrylamide solution into a 1000 mL measuring flask and diluted with deionized water. Three millilitres of the sample supernatant (or standard solution) were derivatized in a glass sample tube by adding 1.5 g of potassium bromide (KBr), 1 mL of potassium bromate solution (0.1 M, $KBrO_3$) and 0.3 mL of sulfuric acid solution (50%, H_2SO_4). The mixture was mixed in a shaker and kept for 2 h in a refrigerator (~4 °C). The derivative was neutralized by adding 250 µL of sodium thiosulfate solution (1 M, $Na_2S_2O_3x5H_2O$) until the orange colour disappears. Approximately 1.5 g of sodium chloride (NaCl) was added to the derivatization mixture and the mixture was extracted with ethyl acetate ($CH_3COOC_2H_5$) (2 x 5 mL). The collected ethyl acetate was concentrated with a rotary evaporator system (Christ CT 02-50, Germany) at a temperature of 40 °C and under reduced pressure. The solvent was evaporated and dissolved in 0.5 mL of ethyl acetate (for the standard, in a volume of 3 mL). One hundred milligrams of anhydrous sodium sulphate (Na_2SO_4), 20 µL of triethylamine [$(C_2H_5)3N$] (20 µL of triethylamine in 0.5 mL of a concentrated derivatization solution) was added to the solution in a 15 mL centrifuge tube, mixed and centrifuged for 10 minutes (4000 rpm). The supernatant was analysed with a gas chromatograph – electron capture detector (GC–ECD). To that purpose, a gas chromatograph (Shimadzu GC-17A, Japan) was equipped with an electron capture detector and an integrator to measure peak areas, and a thermostated column. The capillary column was a Rxi-5Sil MS (Restek, Germany) with 30 m length x 0.25 mm-ϕ inner diameter and 0.25 µm-ϕ stationary phase film thickness. Working conditions were: injection volume 1 µL; column temperature gradient 70 °C (hold 1 min), 3 °C/min to 140 (hold 0.5 min), 15 °C/min to 280 (hold 4 min). The mobile phase was nitrogen at 18.0 cm/sec flow-rate, with a split of 3.0. The injector temperature was 250 °C, the detector temperature was 260 °C and the detector current was 2 nA.
